# Cancer and Thrombosis: The Platelet Perspective

**DOI:** 10.3389/fcell.2016.00147

**Published:** 2017-01-05

**Authors:** Claire K. S. Meikle, Clare A. Kelly, Priyanka Garg, Leah M. Wuescher, Ramadan A. Ali, Randall G. Worth

**Affiliations:** Department of Medical Microbiology and Immunology, University of Toledo College of Medicine and Life SciencesToledo, OH, USA

**Keywords:** platelet, metastasis, thrombosis, cancer, TCIPA

## Abstract

Platelets are critical to hemostatic and immunological function, and are key players in cancer progression, metastasis, and cancer-related thrombosis. Platelets interact with immune cells to stimulate anti-tumor responses and can be activated by immune cells and tumor cells. Platelet activation can lead to complex interactions between platelets and tumor cells. Platelets facilitate cancer progression and metastasis by: (1) forming aggregates with tumor cells; (2) inducing tumor growth, epithelial-mesenchymal transition, and invasion; (3) shielding circulating tumor cells from immune surveillance and killing; (4) facilitating tethering and arrest of circulating tumor cells; and (5) promoting angiogenesis and tumor cell establishment at distant sites. Tumor cell-activated platelets also predispose cancer patients to thrombotic events. Tumor cells and tumor-derived microparticles lead to thrombosis by secreting procoagulant factors, resulting in platelet activation and clotting. Platelets play a critical role in cancer progression and thrombosis, and markers of platelet-tumor cell interaction are candidates as biomarkers for cancer progression and thrombosis risk.

## Introduction

Platelets were first described as an independent cell type present in the blood in 1881 by Giulio Bizzozero (reviewed in Mazzarello et al., 2001). They were named after their morphology in the non-activated state, small discoid cells resembling “small plates.” Bizzozero was also the first to describe the morphological changes in platelets attributed to platelet activation and their important role in thrombus formation (Bizzozero, 1881). Subsequently, James Homer Wright described platelets as originating from megakaryocytes in the bone marrow (Wright, 1910). Many bleeding disorders and diseases attributed to defects in platelet function were described during this period.

Platelets are produced by hematopoietic stem cells in the liver during fetal development and can be seen in fetal circulation as early as 8–9 weeks (Palis and Segel, 2016). Platelets quickly reach adult quantities, and neonatal thrombocytopenia is defined by the same criteria as adult thrombocytopenia (<150 × 10^9^/L) (Sola-Visner, 2012). Late in gestation through adulthood, platelet production shifts to megakaryocytes (MKs) in the bone marrow. Once mature, MKs migrate to the vascular bed and release proplatelets into the circulation, eventually leading to the dissolution of the entire MK (Machlus and Italiano, 2013). Once in circulation, proplatelets break apart and form mature platelets that travel through the circulation for 7–10 days before being cleared by resident phagocytes in the liver and spleen (Sørensen et al., 2009).

### Platelet composition

Platelets possess and display a variety of functional immunoreceptors that respond to a broad spectrum of agonists including those associated with tissue injury and infection (Kasirer-Friede et al., 2007; Cox et al., 2011). Platelets have glycoproteins (GPs) that sense vascular and sub-endothelial structures such as collagen and other exposed proteins, which enable platelets to respond to injury. GPIbα and GPVI are involved in thrombus formation (Gardiner and Andrews, 2014), and GPIIb/IIIa, also known as integrin αIIbβ3, is critical for platelet aggregation, adhesion to ECM, and clot retraction (Kasirer-Friede et al., 2007). Platelets also express pattern recognition receptors (PRRs) including toll-like receptors (TLRs), NOD-like receptors (Zhang S. et al., 2015), and C-type lectin receptors (Polgar et al., 1997). TLR expression enables activated platelets to bind and capture bacteria (Cognasse et al., 2005; Aslam et al., 2006). Other important surface receptors including P-selectin (Furie et al., 2001), integrins (Kasirer-Friede et al., 2007; Bennett et al., 2009), and FcγRIIa (Berlacher et al., 2013) also increase upon platelet activation, facilitating interactions between activated platelets and leukocytes.

Platelets are continually exposed to all components of plasma through their open canalicular system, which provides a conduit for release of granule contents, and facilitates platelet shape change in response to stimuli (Escolar et al., 1989; Escolar and White, 1991; Jurk and Kehrel, 2005). Mature platelets possess three distinct types of cytoplasmic storage compartments: alpha (α-) granules, dense (δ-) granules, and lysosomal (λ-) granules. These granules contain vast array of bioactive molecules with hemostatic and host defense properties that can be released into the circulation or translocated to the platelet surface upon platelet activation. α-granules are the most abundant type and contain bioactive mediators including adhesion molecules, coagulation factors, growth factors, cytokines and chemokines, and microbicidal proteins. δ-granules store bioactive amines (histamine and serotonin), ions (calcium and phosphate), and nucleotides (ADP and ATP) (Yeaman, 2014). The list of proteins housed in each type of granules is summarized in our previous report (Ali et al., 2015).

Platelets are anucleate, but they contain thousands of unique RNA transcripts (Zimmerman and Weyrich, 2008), including long-lived mRNA that can act as a template for protein translation throughout the platelet lifespan (Booyse and Rafelson, 1967) and unspliced pre-mRNA that can be processed by megakaryocyte-derived spliceosome (Denis et al., 2005). In addition to translation mechanisms common to many cell types, platelet activation influences translation via mTOR signaling (Weyrich et al., 1998). The 5′ and especially 3′ untranslated regions of platelet mRNA contribute to differential translation and transcript half-life (Zimmerman and Weyrich, 2008), and activated-platelet protein-1 expressed during platelet activation binds poly A sequences in the 3′ region to regulate translation (Houng et al., 1997). Notably, platelet activation stimulates translation of multiple proteins (Denis et al., 2005) including Bcl-3 (Weyrich et al., 1998), tissue factor (Schwertz et al., 2006), and IL-1β (Lindemann et al., 2001), among many others.

In addition to mRNA, platelets also contain microRNAs (miRNAs). miRNAs are small non-coding RNAs shown to play important roles in gene regulation, and act as biomarkers and regulators of disease states (Ardekani and Naeini, 2010; Li and Kowdley, 2012; McManus and Freedman, 2015). Platelets possess the machinery necessary for processing pre-miRNA to functional miRNA (Landry et al., 2009). Platelets contain a distinct miRNA profile and changes to miRNA within the platelet can lead to dysfunctional platelet activity (Plé et al., 2012; Rowley et al., 2016). Platelet miRNA can also exert effects on surrounding tissues, leading to decreased expression of intercellular adhesion molecule-1 (ICAM-1) on the endothelium during myocardial infarction (Gidlöf et al., 2013). As they are abundant in the circulation and are able to secrete bioactive miRNA that can affect surrounding cells and tissues, platelets' role in regulation of health and disease can be significant.

### Platelet function

Platelets play a critical role in hemostasis and immunity, and are among the first cells to detect endothelial injury and microbial pathogens invading the bloodstream and tissues (Al Dieri et al., 2012; Gardiner and Andrews, 2013). Platelets circulate in a quiescent state without forming stable adhesions with the endothelial cells. Vascular injury causes platelet glycoproteins GPVI and GPIbα to interact with exposed collagen and von Willebrand Factor (VWF), respectively, in the subendothelial matrix. These receptor-ligand interactions mediate platelets' stable adhesion to the endothelial cells and initiate a cascade of intracellular responses that results in amplification of activation signals through the release of platelet agonists like ADP and thrombin. In response to activation, platelets change their shape, degranulate, and upregulate surface receptor expression. Collectively, this leads to further platelet aggregation and recruitment to the sites of tissue damage or infection (Semple et al., 2011). In addition to activation by classic platelet agonists, platelets can also be partially activated or “primed” by the presence of atypical agonists such as IgG (Antczak et al., 2010). Human platelets possess the Fcγ receptor IIa that is actively able to bind and internalize IgG complexes (Worth et al., 2006; Antczak et al., 2011). Pre-stimulation with IgG complexes leads to increased activation in response to lower levels of agonists causing a state of “platelet hypersensitivity” (Berlacher et al., 2013). We have shown this phenomenon in systemic lupus erythematosus (SLE), an inflammatory condition that is known to have circulating IgG complexes.

## Platelet interaction with immune cells with respect to cancer

Platelets play a role in inflammation by binding to immune cells to modulate immune function. Binding of activated platelets to leukocytes stimulates cytokine release, oxidative burst, phagocytosis, and formation of neutrophil extracellular traps (NETs), which are composed of DNA, histones, and antimicrobial proteins (Kral et al., 2016). Platelets also recruit and activate macrophages and neutrophils in tumor tissue, stimulating TGF-β release and platelet-tumor cell aggregation (Kim and Bae, 2016). Macrophages infiltrate tumors and release cytolytic factors including tumor necrosis factor α (TNF-α) to destroy the tumor (Larrick and Wright, 1990), and it was found that platelets reduced macrophage-mediated cytotoxicity in fibrosarcoma by inhibiting the effects of TNF-α (Philippe et al., 1993). Immune cells may also stimulate platelets. For example, neutrophil release of myeloperoxidase partially activates platelets (Kolarova et al., 2013). Moreover, NETs stimulate the intrinsic pathway of the coagulation cascade, ultimately generating thrombin and activating platelets (Gould et al., 2014).

Platelet-immune cell interactions have applications in cancer treatment. For example, elevated PLR correlated with elevated CA 19-9, the current biomarker used of diagnosis of pancreatic cancer (Miglani et al., 2013). Similar changes in PLR have also been reported in patients at risk of lung cancer (Sanchez-Salcedo et al., 2016). Therefore, changes in PLR are most likely not cancer-specific and may not be able to differentiate specific cancer types based solely on PLR levels. However, elevated PLR is reported as a prognostic tool (Zhou et al., 2014; Cummings et al., 2015; Messager et al., 2015; Zhang Y. et al., 2015; Wang et al., 2016) and staging and follow up tool (Emir et al., 2015; Jia et al., 2015), often in conjunction with neutrophil/lymphocyte ratio (NLR). Furthermore, PLR has been found to be an independent predictor of venous thromboembolism in cancer patients (Ferroni et al., 2015). COmbination of Platelet count and Neutrophil to Lymphocyte Ratio (COP-NLR) (Ishizuka et al., 2013, 2014; Feng et al., 2014a; Zhang H. et al., 2015; Nakahira et al., 2016) and Neutrophil-Platelet Score (NPS) (Watt et al., 2015) are predictors of survival for several types of cancer. Notably, some scoring systems that include evaluation of platelet count or function have higher predictive value in certain cancers than tools not analyzing platelets (Feng et al., 2014b; Ferroni et al., 2015; Sanchez-Salcedo et al., 2016), indicating that platelets may play a key role in cancer development and have prognostic value.

## Platelets and cancer progression

### Platelet-tumor cell interaction

Platelets play an integral role in the development and metastasis of cancer; high platelet counts have been linked to increased metastasis (Buergy et al., 2012) and poorer outcomes in multiple types of cancer (Kim et al., 2014, 2015; Moschini et al., 2014; Voutsadakis, 2014; Chadha et al., 2015; Ji et al., 2015). Tumor cells interact with platelets through a number of receptors and signaling molecules. For example, tumor cells release soluble molecules that activate platelets, including ADP and thrombin (Zucchella et al., 1989; Boukerche et al., 1994; Bambace and Holmes, 2011) while direct contact of platelets with tumor cells also results in activation (Suzuki-Inoue et al., 2006; Erpenbeck and Schon, 2010; Lal et al., 2013; Menter et al., 2014; Li, 2016). Platelet-tumor cell aggregates form through binding of platelet integrin α_IIb_β_3_ to tumor cell integrin α_v_β_3_ via RGD-containing proteins including fibrinogen, von Willebrand factor, and fibronectin (Kitagawa et al., 1989; Felding-Habermann et al., 1996), a process known as tumor cell-induced platelet aggregation (TCIPA) (Jurasz et al., 2004; Goubran et al., 2013). electins expressed on platelets, leukocytes, and endothelium may also bind tumor cells to form platelet-tumor-leukocyte aggregates (Laubli and Borsig, 2010). Specifically, P-selectin expressed on the surface of activated platelets binds to many types of human cancer cells (Chen and Geng, 2006). Activated platelets were observed to interact with small cell lung cancer and neuroblastoma cell lines, and this interaction was blocked with inhibitory anti-P-selectin antibodies (Stone and Wagner, 1993), indicating that P-selectin is a key mediator of platelet-tumor interaction (Borsig, 2008). L-selectin on leukocytes acts synergistically with P-selectin, facilitating platelet-tumor interaction (Borsig et al., 2002). Podocalyxin-like protein 1 (PCLP1) binds E- and L-selectin in pancreatic cancer (Dallas et al., 2012), and is overexpressed in many cancers and on activated platelets (Amo et al., 2014). Once activated, platelets can then bind to tumor cells via P-selectin (Chen and Geng, 2006; Coupland et al., 2012; Qi et al., 2015) and glycoproteins (Lonsdorf et al., 2012; Goubran et al., 2013) and directly induce tumor growth and metastasis by releasing pro-tumor angiogenic and growth factors.

Platelet-derived microparticles (PDMPs) also promote metastasis and angiogenesis by producing matrix metalloproteinase 2 (MMP-2), angiogenic platelet-derived growth factors and tissue factor (Dashevsky et al., 2009; Martinez and Andriantsitohaina, 2011; Falanga et al., 2012; Varon et al., 2012). These pathways create a loop of activation as tumor cells activate platelets, which in turn support growth, invasion, and metastasis of tumor cells (Goubran et al., 2013).

Tumor cells transfer RNA into platelets via microvesicles, resulting in tumor educated platelets (TEPs) (Nilsson et al., 2011). Importantly, this observation was applied to cancer diagnostics using mRNA sequencing of TEPs. Sequencing of TEPs was 96% accurate in distinguishing cancer patients from individuals without cancer, and was able to provide information about the location of some cancers (Best et al., 2015). Platelet mRNA profiles showed downregulation of numerous genes, including many associated with translation, IL-signaling, protein synthesis, and immunity, and upregulation of cancer-associated, metabolic, cytoskeletal, and platelet-related genes (Best et al., 2015). EML4-ALK rearrangements in non-small-cell lung cancer were detectable by RT-PCR in platelets, and this correlated with poor prognosis (Nilsson et al., 2015), suggesting TEP may be a promising source for liquid biopsy (Feller and Lewitzky, 2016; Perez-Callejo et al., 2016).

### Platelet effect on tumor invasion and intravasation

Activated platelets play multiple roles in the progression of tumor metastasis, including facilitation of tumor cell epithelial-mesenchymal transition (EMT), degradation of surrounding extracellular matrix (ECM), increasing vascular permeability, and aiding in the establishment of malignancies in distant tissues (Miyashita et al., 2015; Pang et al., 2015; Guillem-Llobat et al., 2016) through interactions with tumor cells through selectins and glycoproteins (Kohler et al., 2010; Laubli and Borsig, 2010; Gay and Felding-Habermann, 2011; Bendas and Borsig, 2012; Pang et al., 2015). Activated platelets promote metastasis by secreting lysophosphatidic acid (LPA), a lipid that has growth factor-like properties, which has been found to be involved in the progression of multiple cancers (Mills and Moolenaar, 2003; Leblanc and Peyruchaud, 2015; Lou et al., 2015). LPA plays a role in many cellular processes including cell proliferation, survival, migration, tumor cell invasion, and reversal of differentiation through multiple G protein-coupled receptor (LPA1-6) cascades, and is a potential target for cancer therapy (summarized in Mills and Moolenaar, 2003). Activated platelets also secrete transforming growth factor β (TGF-β) (Assoian and Sporn, 1986) and platelet-derived growth factor (PDGF) (Kong et al., 2008) from α-granules in response to tumor cell stimulation, inducing EMT (Assoian and Sporn, 1983; Radisky and LaBarge, 2008; Labelle et al., 2011; Yu et al., 2013; Leblanc and Peyruchaud, 2016). By releasing TGF-β and PGE2, platelets strongly activate genes promoting EMT, ECM remodeling, and metastasis in tumor cells (Labelle et al., 2011; Guillem-Llobat et al., 2016). Tumor cell expression of the EMT-associated transcription factor Snail1 correlated with platelet localization on the leading edge of tumor cells as indicated by CD42b (Miyashita et al., 2015). This is an important area of investigation that may yield important results.

Platelets also influence tumor metastasis by enhancing tumor cell expression of tissue factor, a primary initiator of the coagulation cascade (Orellana et al., 2015). Tissue factor is constitutively present on the surface of malignant tumors which activates platelets (Callander et al., 1992; Date et al., 2013), and is shed on tumor microvesicles (Yu and Rak, 2004). Tissue factor generates thrombin, which has been shown to induce platelet-tumor cell interactions *in vitro*, and administration of thrombin increases pulmonary metastases in murine models of colon cancer (summarized in Gay and Felding-Habermann, 2011). Tissue factor also drives growth of primary tumors, stimulates angiogenesis, and is associated with EMT and cancer stem cell behavior (Versteeg, 2015), and was found to be an indicator of metastasis and prognosis in numerous types of cancer (summarized in van den Berg et al., 2012).

Once tumor cells undergo EMT, the next step in metastasis is to invade local tissues and enter the bloodstream (Hunter et al., 2008). Tumor cells activate platelets through a number of mechanisms, including release of platelet-activating soluble factors like ADP and thrombin and ligation of TLR-4 (Grignani et al., 1989; Li, 2016). Activated platelets then release serotonin, ATP and histamine increasing vascular permeability, and MMPs that degrade ECM (Deryugina and Quigley, 2006; Li, 2016). Platelet-derived LPA also up-regulates matrix metalloproteinase (MMP) activity (Lou et al., 2015). The weakened extracellular matrix and endothelial barrier allow tumor cells to enter circulation and metastasize (Stegner et al., 2014).

### Tumor shielding

Tumor cells free in circulation are susceptible to immune surveillance and killing. Natural killer (NK) cells are the primary killers of metastasizing tumor cells (Talmadge et al., 1980; Wiltrout et al., 1985). Platelets shield tumor cells from NK cell lysis by forming aggregates on the tumor cell surface (Nieswandt et al., 1999). Platelets interfere with NK cell binding to tumor cells both sterically and by inhibiting NK cell cytolytic function (Philippe et al., 1993; Shau et al., 1993). Specifically, activated platelets release soluble factors (e.g., TGF-β) that down-regulate NK cell immunoreceptors and inhibit NK cell functions including IFN-γ production, cytotoxicity, and granule mobilization (Kopp et al., 2009).

Platelets also may facilitate tumor escape from NK cell lysis by modulating expression of major histone compatibility complex (MHC) class I. MHC class I is an antigen that host cells express to identify them as “self” or to present antigen fragments to CD8^+^ T cells if the host cell is infected or abnormal. Many types of malignancies have been shown to express abnormal MHC class I, including MHC class I with structurally altered heavy chains, mutated β_2_-microglobulin and TAP1, or dysregulated antigen processing machinery, leading to reduced or absent MHC class I expression (Seliger, 2008, 2014). This dysregulation makes tumor cells susceptible to killing by NK cells, which target cells lacking MHC class I (Seliger, 2008). Platelets can transfer MHC class I antigens to tumor cells, protecting them from T-cell mediated immunity without inducing NK cell cytotoxicity and IFN-γ production (Placke et al., 2011, 2012), and direct platelet inhibition of tumor cell lysis by NK cells can also occur in an MHC-independent manner (Nieswandt et al., 1999). In mice, thrombocytopenia inhibits metastasis but this effect is reversed if NK cells are depleted, indicating platelets' key role in metastasis is shielding tumor cells from NK cell killing (Kopp et al., 2009). Platelets and platelet-derived microparticles adhere to tumor cells through interactions with integrins and selectins (Kitagawa et al., 1989; Felding-Habermann et al., 1996; Gay and Felding-Habermann, 2011). Additionally, fibronectin and other adhesive molecules may act as a bridge between platelets and tumor cells, as mediated by PCLP1 (Amo et al., 2014). Tumor cells are enshrouded in platelet-fibrin mesh, shielding them from NK cell contact and immune surveillance in circulation (Borsig et al., 2001).

### Platelet effect on tumor cell arrest and extravasation

Platelets and platelet-derived microparticles adhered to circulating tumor cells also facilitate tethering and arrest (Honn et al., 1992; Gay and Felding-Habermann, 2011; Menter et al., 2014; Li, 2016). Platelets were found to act as chemoattractants for circulating tumor cells, potentially aiding in tumor cell homing and establishment of metastatic sites (Orellana et al., 2015). Tumor cell-activated platelets release ATP, which binds P2Y_2_ receptors on the endothelium, opening the transendothelial barrier and allowing tumor cells to exit the bloodstream into metastatic sites (Schumacher et al., 2013). LPA, TGFβ, MMP, PGE_2_, and other platelet- and leukocyte-derived factors that assisted in EMT and intravasation weaken the endothelium and also facilitate extravasation (Stegner et al., 2014). Platelet-bound tumor cells may bind directly to selectins on the endothelium, leading to tethering, rolling, and ultimately extravasation (Bendas and Borsig, 2012; Coupland et al., 2012). Myeloid cells can facilitate this process by activating the endothelium through interleukin (IL)-1α, IL-1β, and TNF-α (Labelle and Hynes, 2012). Tumor cells may also be slowed due to their large size upon reaching microvasculature, among other mechanisms (summarized in Witz, 2008). Notably, the pro-coagulant characteristics of platelet-tumor aggregates facilitate the formation of microthrombi in small vessels, further slowing tumor cell migration and enhancing arrest rate (Menter et al., 2014).

### Platelet effect on tumor cell establishment and angiogenesis

Tumor cells do not necessarily remain and grow at the initial site of arrest; many cells die or become dislodged, and some cells have been observed to leave and reattach at another site (Kienast et al., 2010). Successful metastasis requires extravasation close to sufficient vasculature to allow tumor cells to obtain nutrients (Folkman, 1971) and recruit leukocytes to form premetastatic niches (Labelle and Hynes, 2012). Premetastatic niche formation depends on communication with the microenvironment (LaBarge et al., 2007; LaBarge, 2010), including platelet-derived TGF-β and P2Y_12_ signaling (Labelle et al., 2011; Wang et al., 2013). TGF-β reduces the effect of tumor-entrained neutrophils (TEN; a subset of CD11b^+^Ly-6GH^+^MMP-9^+^ cells not present in healthy individuals) (Fridlender et al., 2009), which typically kill tumor cells by producing hydrogen peroxide (Granot et al., 2011).

Platelet-activated tumor cells have enhanced expression of pro-metastatic genes including proteases, cytokines, and growth factors to facilitate invasion and metastatic seeding (Labelle et al., 2011). Among other pro-angiogenic factors (summarized in Sabrkhany et al., 2011), platelets are a primary source of vascular endothelial growth factor (VEGF), a growth factor that increases vascular permeability, promotes extravasation, and is critical for angiogenesis (Verheul et al., 1997; Sierko and Wojtukiewicz, 2004). Platelets activated by TF present on the surface of endothelial cells (Shoji et al., 1998; Verheul et al., 2000) release VEGF in malignant tissue, directly promoting angiogenesis (Verheul et al., 2000). Tumor-derived thrombin also plays an angiogenic role, inducing endothelial cell growth (Herbert et al., 1994) and increasing platelet release of VEGF. Platelets are thought to contain angiogenesis stimulators and inhibitors secreted based on stimulation of proteinase activated receptors (PARs). Some studies show angiogenesis stimulators and inhibitors are differentially released in response to selective PAR agonists to regulate angiogenesis (Ma et al., 2005; Italiano et al., 2008). However, kinetic analysis revealed no functional pattern in α-granule release, and both types of releasates were ultimately shown to stimulate angiogenesis *in vitro* and *in vivo* (Jonnalagadda et al., 2012; Huang et al., 2015).

Platelet microparticles also promote angiogenesis (Martinez and Andriantsitohaina, 2011), stimulating the formation of capillary tube and network formation (Kim et al., 2004; Prokopi et al., 2009) and stimulating tumor cell expression of pro-angiogenic factors (Janowska-Wieczorek et al., 2005). Microparticles in cancer patients also express tissue factor (Hron et al., 2007), further activating platelets and stimulating the angiogenic cascade to support metastatic tumor growth.

## Cancer and coagulation

The presence of tumor cell-activated platelets in the bloodstream may predispose cancer patients to thrombotic events. The correlation between cancer and risk of venous thromboembolism (VTE), first noted in 1865 by Dr. Armand Trousseau, has been well-documented (Varki, 2007). A 2007 study found that cancer patients on chemotherapy were 47 times more likely to experience VTE (Khorana et al., 2007). In general, active cancer increases risk for VTE by four- to seven-fold, and cancer-associated VTE is on the rise (Key et al., 2016). Cancer patients with elevated pre-chemotherapy platelet counts were significantly associated with VTE, indicating that platelets likely play a role in the development of thrombotic events (Khorana et al., 2005; Kadlec et al., 2014).

Tumor cells stimulate clotting and thrombosis through multiple mechanisms (Mackman, 2008). Tissue factor produced by tumor cells and tumor-derived microparticles stimulates the coagulation cascade (Owens and Mackman, 2011; Menter et al., 2014; Phillips et al., 2015), activating platelets and promoting thrombosis via the tissue factor (extrinsic) pathway (Mackman, 2008). Indeed, plasma tissue factor was found to be predictive of VTE in pancreatic cancer (Khorana et al., 2008). Activated platelets and negatively-charged phospholipids shed by tumor cells may also stimulate coagulation via the contact activation (intrinsic) pathway (Dicke and Langer, 2015; Key et al., 2016). Tumor-derived IL-6 and hepatic thrombopoietin were also associated with thrombocytosis and thrombosis (Hisada et al., 2015). Additionally, tumor-derived microparticles have strong procoagulant activity and are associated with VTE in cancer (Manly et al., 2010; Geddings and Mackman, 2013; Mege et al., 2016). These microparticles were found to activate platelets and induce aggregation and thrombus formation, and accumulated in the thrombus by interacting with P-selectin (Thomas et al., 2009), demonstrating the integral role platelets play in the development of cancer-associated VTE.

VTE and thrombocytosis are factors associated with poor prognosis for patients with cancer (Sorensen et al., 2000; Kourelis et al., 2014; Chadha et al., 2015; Chen et al., 2015), highlighting the need for a method to quantify VTE risk in cancer patients. Markers of platelet-tumor cell interaction including P-selectin, tissue factor, and microparticles are candidates to detect early signs of VTE, as are markers of inflammation (C-reactive protein) and coagulation (D-dimer, Factor VIII; summarized in Hanna et al., 2013). Early detection of risk factors associated with VTE may influence use of thromboprophylaxis, and may substantially reduce morbidity and mortality in cancer patients. Given their critical role in tumor growth, metastasis, and cancer-associated thrombosis, markers of platelet activity should be explored as biomarkers and potential therapeutic targets for cancer progression and VTE.

Platelets play a critical role in cancer progression and metastasis, and contribute to the development of VTE in cancer. However, it is not yet clear whether platelet activation and thrombocytosis are ultimately the causative agent or the result of tumor progression. Additionally, the molecular mechanism behind platelet-induced coagulation in cancer has yet to be described. As research continues to utilize platelet biomarkers in cancer diagnosis, prognosis, and risk, much will be discovered about the platelet-cancer dynamic on a mechanistic level.

## Author contributions

CM, CK, PG, LW, and RA wrote sections of the manuscript. CM and RW compiled and organized the manuscript and RW edited the manuscript.

### Conflict of interest statement

The authors declare that the research was conducted in the absence of any commercial or financial relationships that could be construed as a potential conflict of interest.
